# Automated Discrimination of Brain Pathological State Attending to Complex Structural Brain Network Properties: The Shiverer Mutant Mouse Case

**DOI:** 10.1371/journal.pone.0019071

**Published:** 2011-05-27

**Authors:** Yasser Iturria-Medina, Alejandro Pérez Fernández, Pedro Valdés Hernández, Lorna García Pentón, Erick J. Canales-Rodríguez, Lester Melie-Garcia, Agustin Lage Castellanos, Marlis Ontivero Ortega

**Affiliations:** 1 Neuroimaging Department, Cuban Neuroscience Center, La Habana, Cuba; 2 Basque Center on Cognition Brain and Language, Donostia-San Sebastián, Basque Country, Spain; 3 Laboratory of Cognitive Neuroscience, Universidad Diego Portales, Santiago, Chile; 4 Neurostatistic Department, Cuban Neuroscience Center, La Habana, Cuba; 5 Centro de Investigacion Biomedica en Red de Salud Mental (CIBERSam), Madrid, Spain; 6 Benito Menni Complex Assistencial en Salut Mental, Barcelona, Spain; 7 Neuroengineering Department, Cuban Neuroscience Center, La Habana, Cuba; Beijing Normal University, China

## Abstract

Neuroimaging classification procedures between normal and pathological subjects are sparse and highly dependent of an expert's clinical criterion. Here, we aimed to investigate whether possible brain structural network differences in the shiverer mouse mutant, a relevant animal model of myelin related diseases, can reflect intrinsic individual brain properties that allow the automatic discrimination between the shiverer and normal subjects. Common structural networks properties between shiverer (C3Fe.SWV Mbp^shi^/Mbp^shi^, n = 6) and background control (C3HeB.FeJ, n = 6) mice are estimated and compared by means of three diffusion weighted MRI (DW-MRI) fiber tractography algorithms and a graph framework. Firstly, we found that brain networks of control group are significantly more clustered, modularized, efficient and optimized than those of the shiverer group, which presented significantly increased characteristic path length. These results are in line with previous structural/functional complex brain networks analysis that have revealed topologic differences and brain network randomization associated to specific states of human brain pathology. In addition, by means of *network measures spatial representations* and discrimination analysis, we show that it is possible to classify with high accuracy to which group each subject belongs, providing also a probability value of being a normal or shiverer subject as an individual anatomical classifier. The obtained correct predictions (e.g., around 91.6–100%) and clear *spatial* subdivisions between control and shiverer mice, suggest that there might exist specific network *subspaces* corresponding to specific brain disorders, supporting also the point of view that complex brain network analyses constitutes promising tools in the future creation of interpretable imaging biomarkers.

## Introduction

Complex brain network analysis, in which the brain is modeled as a graph whose nodes (or vertices) represent structural/functional regions and the links (or edges) between them represent anatomical or functional connections, provide us with topological measurements that could be interpreted in terms of the management and integration of the nervous information flow and physiological brain dynamics. Initial analyses of brain networks in the graph framework were devoted to describe the key organizational principles of the normal brain, reporting certain brain topological features such as high clustering, small-worldness, the presence of highly connected hubs, assortativity, modularity or hierarchy, properties that are not typical of random graph and regular lattices (for a review see [1]). However, current trends in brain networks analyses are more focused to detect differences in particular topologic measures associated to specific human states of pathology, such as Multiple Sclerosis (MS) [2], tumors [3], Alzheimer's disease [4], [5], Schizophrenia [6], [7] and Stroke [8], [9], contributing to the understanding of pathophysiological mechanisms, and supporting in general the hypothesis that network randomization and subsequent loss of optimal organization could be a common final result of the brain's reaction to lesions or neurodegenerative processes [8].

Among the diversity of techniques from which brain networks could be extracted [10]–[21], DW-MRI techniques are promising in particular to evaluate topological differences in those brain disorders where the white matter is severely affected, like Multiple Sclerosis [22], [23] and Acute Disseminated Encephalomyelitis [24], [25]. Based in the non-invasive acquisition of structural information about the intravoxel axons arrangement, DW-MRI techniques allows the *in vivo* approximate mapping of the brain nervous fiber circuitry [17], [26]–[31]. However, despite of the demonstrated usefulness of DW-MRI techniques to detect anomalies [32]–[38], to date, brain network analyses based on DW-MRI tractography techniques have been more devoted to describe the brain organizational principles described above [15], [17], [18], [39]–[42] than to the study of specific brain pathologies [5], [9], [43], limiting its potential applications to the quantitative description and understanding of specific brain disorders, something with a possible practical outcome for clinical diagnosing.

Here, in line with previous pathophysiological brain studies in a graph framework, we propose to search for altered topological properties using fiber tractography DW-MRI applied to a brain disease where the white matter is severely affected. We have the further purpose of investigating whether possible brain structural network differences reflect intrinsic individual brain properties that allow the automatic discrimination between pathological and normal subjects. More specifically, we search for altered topological properties in six different basic parameters (i.e. clustering, characteristic path length, modularity, global/local efficiency and small-worldness) in the shiverer mouse, a mutant model relevant to the study of myelin related diseases since it is characterized by a deletion of the gene encoding myelin basic protein (MBP), resembling white matter dysmyelinating and demyelinating process that takes place in humans due to an inflammatory process, for example, in those patients affected by MS [22], [23], [44]. In addition, because a specific focus of clinical diagnostic investigation is the anatomic discrimination between normal and pathological states, we perform an automatic discrimination between shiverer and control subjects based on these complex network characteristics. In order to perform the automatic subject classification, the concept of *network measure spatial representation* is introduced. In this, for each network measure, each subject is spatially represented and determined by a unique point whose coordinates are assigned according to individual network metrics. Then using classification techniques the original *space* is subdivided into two *subspaces,* separating subjects that present similar topological characteristics, and obtaining also an individual probability value of being from one or the other group as an anatomical classifier.

Finally, some comments are made concerning the relationship between the obtained findings and some previously reported human pathological state studies (e.g. MS reports), as well as the possible implications that these complex networks analyses and representations might have on clinical diagnostic investigation, for either, the anatomic classification between normal and pathological states, and the creation of interpretable brain dynamical imaging biomarkers.

## Results

**Figure 1:**
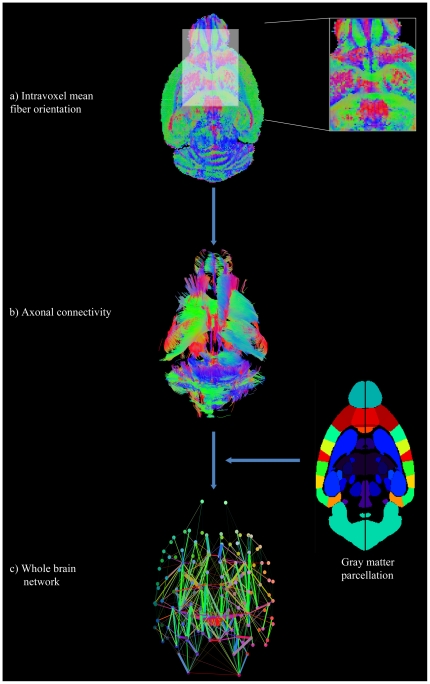
Schematic representation of the connectivity estimation and network construction procedure. Depicted example corresponds to one control subject and FACT tractography algorithm. a) Axial map representing intravoxel mean fiber orientation (dyadic vectors). Inset figure provides detail of the high fiber orientation coherence around the corpus callosum and olfactory areas. b) Obtained whole brain axonal trajectories. c) Whole brain structural network derived as described in *Materials and Methods*; points (nodes) represent anatomic regions, lines (arcs) correspond to connections between them and line widths reflect the corresponding arc weights. In a), b) and c) voxels, fiber trajectories and lines colors were assigned according to the RGB code (i.e. red, green and blue colors indicates rostrocaudal, mediolateral and dorsoventral orientations respectively).

Anatomical connections between cortical and subcortical regions for shiverer (C3Fe.SWV Mbp^shi^/Mbp^shi^, n = 6) and background control (C3HeB.FeJ, n = 6) mice were estimated using three different fiber tractography algorithms applied to data from high resolution DW-MRI (see *Materials and Methods*). From the obtained whole brain axonal trajectories (Figure 1b), weighted networks were created for the whole brain (Figure 1c), in which each node represents an anatomic brain region (150 gray matter regions in total), arcs connecting nodes correspond to white matter links, and arc weights correspond to the degree of evidence supporting the existence of a effective white matter connection between regions. In summary, for each subject we obtained a whole brain network, each one replicated for each of three different fiber tracking algorithms.

### Normal/Shiverer network measures deviation

For these whole brain networks, six different topological properties were evaluated: clustering (*C*, a measure of the inherent tendency to cluster nodes into strictly connected neighbourhoods), characteristic path length (*L*, the average number of region-region direct connections that must be traversed to go from one region to another), modularity (*Q*, the degree to which a network may be subdivided into subnetwork modules with a maximum number of internal links and a minimum number of external links), global efficiency (*E_glob_*, a measure of how much parallel information can be potentially exchanged over a network), local efficiency (*E_loc_*, the average global efficiency of the local subnetworks) and small-worldness (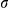, a measure of how optimally is organized a network) (see Table 1).

**Table 1:** Clustering (*C*), characteristic path length (*L*), modularity (*Q*), global efficiency (*E_glob_*), local efficiency (*E_loc_*) and small-worldness (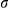) parameters obtained for the brain anatomical networks of control and shiverer mice groups.

Group		Brain network measures(Mean ± SEM)					
		*C*	*L*	*Q*	*E_glob_*	*E_loc_*	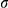
Control	FACT	45.50±1.73	0.06±0.01	0.61±0.00	39.17±0.62	83.49±3.67	5.56±0.23
	TL	46.33±1.28	0.07±0.00	0.65±0.00	37.73±0.37	84.69±2.52	6.31±0.27
	TEND	60.27±2.04	0.06±0.00	0.62±0.00	36.69±0.64	124.22±5.05	5.92±0.22
Shiverer	FACT	32.23±1.92	0.07±0.01	0.59±0.01	29.58±1.52	58.78±4.35	5.47±0.38
	TL	32.71±1.64	0.09±0.01	0.65±0.01	30.01±1.08	62.77±3.85	3.99±0.30
	TEND	47.27±1.41	0.08±0.00	0.60±0.01	29.46±1.14	93.44±2.91	5.07±0.31
**P-value**		**0.0025**	**0.0455**	**0.0324**	**0.0025**	**0.0015**	**0.0005**

For each measure and fiber tracking algorithm, mean values are reported with their corresponding standard errors (i.e. the uncertainty of how the sample mean represents the underlying population mean). For each measure, the multivariate permutation P-value corresponds to the null hypothesis that means of obtained group values are equal (a P-value near to zero, i.e. P<0.05, indicates a significant difference between groups). The small P-values obtained for measures *C, Q, E_glob_* *, E_loc_ and* 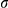 (all P<0.0324) indicates a significant decreases on the shiverer subjects of these structural network attributes, which in conjunction with the significant increase of measure *L* (P<0.05) reflects a considerable reduction in the amount of possible nervous information that can be exchanged over the brain and how deficiently and no optimally it can be managed. For obtained gamma (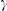) and lambda (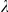) parameters, and their influence on the 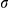 index, please see Table S2. Significant P values are depicted in bold type.

In order to evaluate significant (dis)similarities between the control and shiverer group, for each network measure a multivariate permutation test was performed, testing the null hypothesis of equal means between groups (see *Statistical Analysis* on *Materials and Methods*). We found significant differences for *C* (P = 0.0015), *L* (P = 0.0495), *Q* (P = 0.0324), *E_glob_* (P = 0.0045), *E_loc_* (P = 0.0005) and 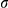 (P<0.0004). For *C*, *Q*, *E_glob_, E_loc_* and 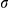 the mean values of shiverer subjects were lowers than the corresponding mean values of control subjects. This indicates a significant reduction of these structural network attributes in the pathological subjects, which in conjunction with the observed significant increase of corresponding *L* values, might be interpreted as a considerable decline in the amount of possible nervous information that can be exchanged over the shiverer's brain, and how deficiently and no optimally it can be managed.

### Subjects classification

**Figure 2:**
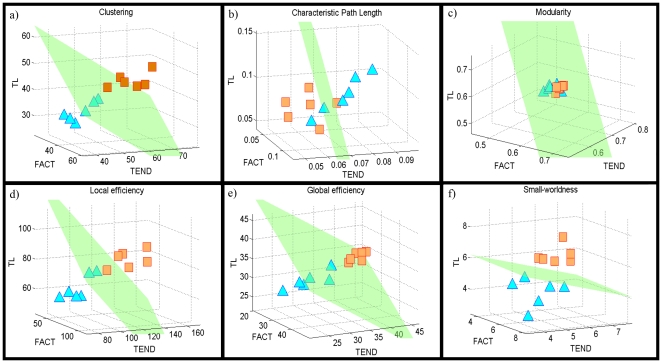
Three-dimensional brain *network measure representation space* for: (a) clustering, (b) characteristic path length, (c) modularity, (d) local efficiency, (e) global efficiency, and (f) small-worldness indices. Control and shiverer subjects are represented by the symbols □and Δ, respectively. For each measure *space*, the green surface constitutes the mean boundary plane between groups obtained by means of a LDA cross-validation approach (see *Subjects Classification* on *Material and Methods* section). Note the correct predictions and clear *spatial subdivisions* between control and shiverer mice for some of the evaluated network measures (panels a, d, e and f), which suggest that might exist specific network *subspaces* corresponding to specific brain disorders.

Figure 2 shows locations of controls and shiverer subjects in the 3-dimensional Euclidian spaces corresponding to the *network measure representation spaces* of *C, L, Q, E_glob_, E_loc_* and 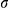
metrics. In each *representation space*, subjects are represented and determined by a unique spatial point, with “length”, “width” and “depth” coordinates assigned according to values obtained from three different fiber tracking algorithms (see *Network Measures Spatial representations* on *Materials and Methods*). To assess the competences of these network topological features to discriminate between groups, linear discriminator analysis (LDA) was used [45](see *Subjects Classification* on *Materials and Methods*). Then for each considered network measure we obtained the mean boundary hyperplane that separated the original *representation space* into two *subspaces,* to which belongs respectively the subjects that presented similar spatial positions (topological properties; see Figure 2). In addition, for each network measure and the combination of all of them we obtained the conditional probabilities of belonging to the identified groups (see Table 2).

**Table 2:** Individual conditioned probabilities of being a control subject with regard to clustering (*C*), characteristic path length (*L*), modularity (*Q*), global efficiency (*E_glob_*), local efficiency (*E_loc_*) or small-worldness (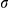) measures obtained for the brain anatomical networks of control and shiverer mice subjects (preceded by the prefixes Wt and Shi, respectively).

Subjects	P(Cs|*C*)	P(Cs|*L*)	P(Cs|*Q*)	P(Cs|*E_glob_*)	P(Cs|*E_loc_*)	P(Cs| 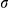 )	P(Cs|*C,L,Q*,*E_glob_* *,E_loc_,* 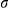 )
Wt 1	0.9999	0.0093	0.8056	0.9999	0.9999	0.9999	0.9999
Wt 2	0.9999	0.6524	0.4438	0.9999	0.9998	0.9560	0.9999
Wt 3	0.9999	0.9724	0.5079	0.9999	0.9999	0.9990	1
Wt 4	0.9999	0.9725	0.9162	0.9999	0.9998	0.9724	0.9999
Wt 5	0.9838	0.7499	0.8080	0.9999	0.8183	0.9722	0.9963
Wt 6	0.9999	0.4841	0.8383	0.9999	0.9999	0.9928	1.0000
Shi 1	0.0179	0.9710	0.7829	0.9889	0.3150	0.0891	0.0083
Shi 2	7.58e-10	0.0163	0.3956	1.97e-10	6.07e-7	2.00e-06	4.44e-16
Shi 3	0.0075	0.1708	0.1862	0.0019	0.1116	0.0327	0.0009
Shi 4	2.30e-10	0.6477	3.2287	2.30e-07	3.31e-06	0.1507	6.66e-16
Shi 5	2.97e-12	0.0339	0.6757	0	7.10e-11	6.93e-05	0
Shi 6	4.46e-07	0.1015	0.6455	5.15e-11	4.19e-06	0.0063	1.87e-12
Predicted (%)	100	66.67	66.67	91.66	100	100	100

For each subject, a P(Cs|I_i_) value near to one, e.g. P>0.95, indicates a high probability of belonging to the control group according to the structural network measure I_i_; whereas a P(Cs|I_i_) value near to zero, e.g. P<0.05, indicates a high probability of belonging to the shiverer group. For comparison, corresponding conditioned probability of being a shiverer subject according to I_i_ can be obtained similarly as 1-P(Cs|I_i_). For each measure, or the combination of all them, the Correct Prediction value indicates the % of subjects that were correctly classified. Note the perfect predictions, i.e. 100 %, obtained from the clustering, local efficiency and small-worldness measures, as well as from the unification of the six considered network measures.

Note the clear *spatial* subdivisions between control and shiverer subjects obtained for *C, E_glob_, E_loc_* and 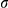, and the corresponding high values of correct predictions (i.e. 100, 91.66, 100 and 100 percent respectively; Table 2), which supports the hypothesis of a possible discrimination between control and pathological (shiverer) subjects based on their brain structural network descriptors. The *representation space* of the *L* and *Q* measures were keep it only for illustrative purposes because, although we previously found a significant difference for these measures, they were not practical to predict between normal and pathological subjects (i.e. providing a low prediction accuracy value of 66.67%, equivalent to predict correctly only 8 of the 12 subjects); as figure 2b and 2c shows the corresponding mean hyperplanes were not able to correctly separate the two groups.

It should be noted also that subject classification based on the combination of the six considered network measures, by means of a forward sequential feature selection in a wrapper fashion (see *Subjects Classification* on *Materials and Methods*), provided 100% prediction accuracy (Table 2). This result it is not surprising when is considered the previously obtained perfect predictions without the combination of all network measures (i.e. aforementioned 100% prediction accuracies for *C, E_loc_* and 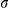 individual measures). However, should be noted that in this case (classification based on the combination of the six considered network measures) the contrast between the obtained individual conditioned probability values of control and shiverer subjects is considerably more accentuated (in the sense of the correct prediction) than when is used only any of the previous measures as a single predictor (Table 2). Thus, the selection of most prominent features [46] allows us to reduce redundant network features information (see Table S6, where is evidenced the characteristic high correlations among almost all the studied network metrics) in order to obtain a final quantitative subject discrimination based on different complementary aspects of the structural brain network.

## Discussion

We performed a structural network analysis based on high resolution DW-MRI techniques and graph theory, to search for altered topological properties in the shiverer mouse using matched healthy mice as controls. We found significant differences for specific network measures such as *C, L, Q, E_glob_* *, E_loc_ and* 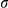, indicating that these metrics (mainly related to the potential amount of nervous information that can be exchanged over the brain, and how efficiently and optimally it could be managed) are significantly altered in the shiverer subjects. In addition, we showed that control and shiverer subjects can be automatically classified by means of *network measures representation spaces* and discriminant analysis (LDA).

### Structural network alterations, correspondence with human pathological studies

The significant reduction on small-worldness parameter that we found here for shiverer subjects is in line with similar reductions reported for human patients of Multiple Sclerosis (MS) [2], which tend to have a smaller number of significant regional cortical thickness correlations and a more randomized structural cortical network organization as the white matter lesion load increases. These results are also in line with changes found in graph theoretical studies of other brain disorders such as tumors [3], Alzheimer's disease [4], Schizophrenia [6], [7] and Stroke [8], which together reinforce the point of view that network randomization and subsequent loss of optimal organization could be a common final result of the brain's reaction to lesions or neurodegenerative processes [8]. In addition, observed significant decreases for global/local structural efficiencies and modularity in the shiverer subjects suggest a lower brain capacity to establish parallel interactions between distant regions as well as a lower tendency to have communities of different anatomical regions that deal with common neural information. As the network extraction methodology used in this study was based on DW-MRI techniques, we can consider that in general these structural differences are directly reflecting variations in the white matter integrity that in the specific case of the shiverer mutant mouse are provoked by dysmyelinating and demyelinating process.

### Subject's classification

The results indicate that is possible to discriminate with high reliability between control and shiverer mice using complex brain structural network properties, providing also a probability of belong to one or the other group as an individual anatomical classifier. Our approach is based on the quantitative differences between network measures (e.g. *C, E_glob_, E_loc_* and 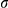) that could be interpreted as reflecting the absence of compact myelin in the central nervous system of shiverer mice. This approach should therefore provide useful information on human brain disorders characterized by dysmyelinating and demyelinating process, like MS.

In the specific case of the MS, in which structural affectations are frequently located in the periventricular and juxtacortical white matter regions, the corpus callosum and infratentorial areas [22], [23], many of traditional diagnostic approaches, as the McDonald criteria [47], needs an expert's intervention as well as subjective tuning parameters, as the required number of T2 lesions (i.e., nine lesions), which makes the diagnostic more difficult and unspecific. However, recent advantages of non conventional MRI techniques such as magnetization transfer, DW-MRI, proton MRI spectroscopy, and functional MRI, have been contributing to overcome the limitations of conventional MRI and associated diagnostic criteria (for a review see [23]). In this sense, possible advantages of novel network analyses as the here proposed is that theoretically allows a deeper understanding of the alterations provoked to the physiological brain dynamics in terms of the management and integration of the nervous information flow. The introduced *network measures representation spaces* concept constitutes an alternative to combine and summarize network topological properties estimated by different modalities (e.g. different fiber tracking algorithms or even different network extraction modalities, like DW-MRI, electroencelography, magnetoencelography and functional MRI). In addition, and although not performed in this study, it is possible to analyze specific nodal properties as an alternative to evaluate problems in specific brain regions and their influence on the whole brain network. Thus, in general the presented approach has potential clinical applications, which in combination with existing criteria might contribute to the future creation of specific brain dynamical imaging biomarkers.

### Methodological issues and future work

Previous methodological studies have provided evidence about how deterministic fiber tracking algorithms can fail on those regions where fibers cross, merge or diverge [48]–[50]. However, our selection of deterministic fiber tracking algorithms was motivated mainly on the fact that the use of high resolution DW-MRI images (80 µm isotropic voxel size) allows a more detailed characterization of the intravoxel anisotropy as well as a considerable reduction of partial volume effects, decreased significantly compared with the high characteristic levels of DW-MRI images acquired at the typical resolutions for which deterministic methods have been traditionally evaluated, e.g. around 2×2×2 mm^3^ (15625 times bigger than the voxel size used here), which even using deterministic tractography algorithms can be translated into a more accurate description of the brain structure. Nevertheless, beyond the statistical nature of the used methods, we emphasize the use of three different tractography algorithms, making the results robust to choice of tracking algorithm, which is potentially a significant source of bias. In addition, the use of *network measures representation spaces* in which results from the different tractography algorithms are represented in the N-dimensional Euclidean space (with an axis corresponding to each tractography algorithm) instead that in a 1-dimensional space (where all tractography algorithms' results are inevitable mixed), allows to apply the discrimination procedure in a way that algorithm interaction effects are reduced, and thus providing a valuable assessment of the relative detail of network information across these methods and a robust set of results with which to assess brain network alterations. Finally, in order to explore quantitatively the performance of the different fiber tracking algorithms with regard the presented discrimination approach, we repeated the subject's classification analysis for each fiber tracking algorithm (see Table S7). As expected, the results confirmed that combination of various fiber tracking algorithms contributed considerably to the stabilization and consistency of the classification results.

A special analysis requires the performed network comparison on a standard brain template. Although individual subjects transformation to the standard template space could introduce propagation of error from normalization procedures, it could be considered that results obtained in a standard space should corresponds mainly to variations on fiber structure and integrity, diminishing possible intersubject fiber tracking variability effects due to technical tracking algorithm limitations, such as the undesired decrease in probability of connection with distance, caused by the progressive dispersion of fiber pathways from voxel to voxel as a consequence of the intrinsic noise and artifacts in the diffusion data. However, in order to explore for differences in subjects classification accuracy with and without transformation to a standard template, we repeated the structural networks construction and subjects classification analysis in the native space (see Table S3). The results indicated a considerable decrease in the prediction accuracy of each network measure and the unification of all them in comparison with previous results obtained on the standard space (Table 2), supporting the point of view that in the case of DW-MRI techniques, subjects transformation to a standard space might allows the improvement of statistical brain network comparisons by reducing variability on networks estimations resultant from technical limitations.

Another consideration for our study is the selection of the mean diffusivity (MD) measure as an indirect measure of changes in potential fiber pathway efficacy in the mouse brain. Other studies have selected with this purpose the *fractional anisotropy* (FA) measure, the number of connecting fiber paths, the MD measure or/and the tensor's three eigenvalues [5], [19], [40], [43]. Our selection was motivated on the fact that MD is a measure of the local average molecular motion, independent of any tissue directionality, which is expected to reflect cellular size and thus fiber integrity [51]–[53]. In that sense, significant decreases of MD (or the diffusion tensor's three eigenvalues) has being reported for many regions of pathological brains characterized by myelin-deficit, at the same time that only a small variation (practically no informative) of other diffusion tensor invariant scalars like FA has being found [44], [54]. However, in order to explore more the arc weight definition used here in comparison to other alternatives, we repeated the structural networks construction and subjects classification analysis firstly using mean FA as a measure of fiber integrity and latter taking arc weight only as the number of fiber connecting paths between any two regions (see Tables S4 and S5, respectively). As expected, the results indicated lower classification accuracies in both cases when compared it to those obtained with the use of MD, although in fact predictions based on mean FA values can be considered as high, particularly for the combination of the 6 considered network measures (i.e. 91.66% of prediction accuracy), supporting the usefulness of define arc weights not only taking into account the basic white matter structure but also the potential efficacy/integrity of each nervous fiber pathway.

Finally, before a potential clinical application can be consider, further studies need to explore mainly two major points: 1) competence of the classification procedure to reflect different levels of lesion profiles and disease states (the pathological subjects that we analyzed here were theoretically at the same brain disorder state, genetically equivalent, and had a mean age at fixation of 6.9±0.2 weeks. This makes it impossible to analyze other factors like temporal progressions or different white matter lesion affectations). Also, 2) reproducibility in human data, which presents different properties referring to images resolution and contrast due to the lower magnetic field strengths that are usually employed in human protocols, i.e. around 1.5–3 Tesla.

## Materials and Methods

### Data acquisition

High-resolution (80 µm isotropic) contrast-enhanced diffusion tensor data was acquired from six background control (C3HeB) and six dysmyelinating shiverer (C3Fe.SWV shi/shi) mouse brains. The data consists of nominally unweighted and diffusion weighted images with optimized icosahedral sampling. This dataset is available as part of the Biomedical Informatics Research Network (BIRN) initiative, accession number TBD, and was downloaded from URL http://www.birncommunity.org/data-catalog/mouse-shiverer-dti-high-resolution-contrast-enhanced-data/(for a related publication see [44]). All experiments were performed in accordance with protocols approved by the Institutional Animal Care and Use Committee of the California Institute of Technology.

#### Animal protocol

The brains of congenic male homozygous shiverer mutants (C3Fe.SWV Mbp^shi^/Mbp^shi^, Jackson Laboratories, mean age at fixation  = 6.0±0.2 weeks, n = 6) and control males with the same background as the shiverers (C3HeB/FeJ, Jackson Laboratories, mean age at fixation 6.9±0.2 weeks, n = 6) were studied using diffusion tensor imaging. Mice were anesthetized deeply using 2.5% Avertin (0.017 ml/g body weight). The mouse was then fixed by transcardiac perfusion using 30 ml of room temperature heparinized phosphate buffered saline followed by 30 ml of room temperature 4% paraformaldehyde (PFA). After death, the head was removed and rocked in 4% PFA overnight at 4C. The skin, lower jaw, ears and cartilaginous nose tip were removed and the head rocked in 50 ml 0.01% sodium azide in PBS for 7.0±0.1 days (mean ± sd) at 4C. The head was then transferred to a 5 mM solution of gadoteridol (Prohance, Bracco Diagnostics Inc, Princeton NJ) and 0.01% sodium azide in PBS and rocked for 13.5±1.9 days at 4C prior to MR imaging. All brains were brought to room temperature for 8.5±3.0 hours immediately prior to imaging at 20C. In four control and four shiverer brains, DTI acquisitions were repeated to address B1 homogeneity concerns and the second dataset used in the results analysis. The additional time spent by these brains in 5 mM gadoteridol is included in the quoted time intervals above. The repeated brains also spent an additional 6.8±0.1 hours equilibrating to room temperature prior to imaging.

#### Image acquisition

All images were acquired using a vertical bore 11.7 Tesla Bruker Avance DRX500 system (Bruker Biospin, Germany) equipped with a Micro2.5 imaging gradient set capable of a peak gradient strength of 1 T/m and a maximum slew rate of 12.5 kT/m/s. The intact head was secured in a Teflon holder and submerged in a perfluoropolyether (Fomblin, Solvay Solexis, Inc, Thorofare, NJ) within a 50 ml vial and imaged using a 35 mm birdcage transmit/receive volume resonator. The ambient bore temperature was maintained at 20C by thermostatically controlled airflow. Optimized second order shimming was achieved across the whole sample using the Bruker implementation of Fastmap 1. Diffusion weighted images were acquired using a conventional pulsed-gradient spin echo (PGSE) sequence (TR/TE  = 150 ms/11.6 ms, 256×150×130 matrix, 19.2 mm ×15 mm ×12 mm FOV, 80 µm isotropic voxel size, 1 average, δ = 3 ms, Δ = 5 ms, Gd = 750 mT/m, nominal b-factor  = 1450 s/mm^2^). An optimized six point icosahedral encoding scheme [55] was used for diffusion weighted acquisitions with a single un-weighted reference image for a total imaging time of 6 hours.

#### Image preprocessing

Individual diffusion tensors maps were estimated [52]. Then, using the Segmentation tools in SPMMOUSE (available at http://www.wbic.cam.ac.uk/~sjs80/spmmouse.html) and SPM5 (available at http://www.fil.ion.ucl.ac.uk/spm/software/spm5/), individual b0 images were non-linearly segmentated into white/gray matter and cerebral spinal fluid probabilistic tissue maps and individual non-linear warping transformation parameters obtained were applied to the corresponding individual diffusion tensors maps [56] in order to transforms them finally into the standard template space of the SPMMOUSE toolbox (a representative atlas of 90 brains scanned at 70 µm isotropic). The previous transformation to the template space was done with the purpose of reduce possible intersubject fiber tracking variability effects on posterior networks estimation and comparison due to technical algorithm limitations, such as the undesired decrease in probability of connection with distance (i.e. the progressive dispersion of fiber pathways with distance that reflects the propagation of uncertainty from voxel to voxel, mainly caused by noise and artifacts in the diffusion data). We comment more about this point in the *Discussion* section.

In addition, we took the image volumes representing the canonical Waxholm Space (WHS) mouse brain [57], which include T1-, T2*-, and T2-Weighted MR volumes, Nissl-stained optical histology, and a label volume describing 37 structures (all volumes are represented at 21.5µ isotropic resolution and are available at http://software.incf.org/software/waxholm-space). From the defined 37 structures we selected 26 gray matter regions. We separated left and right hemispheres, and because in this parcellation scheme the cerebral cortex is originally denoted as only one region, we reparcellated both hemispheric cerebral cortex into 50 small regions of approximately the same volume (1.66±0.23 mm^3^). The previous number of new small cortical regions (i.e., 50 for each hemispheric cortex) was defined trying to ensure on these regions a volume size around the mean volume size of the other considered non-cortical gray matter regions, keeping consequently a minimum volume variation across all considered brain gray matter regions. Then, in order to carry out the hemispheric cerebral cortex parcellation into 50 coherent regions (i.e. non-overlapped regions with a coherent and continue structure), we used the spatial *kmeans* clusterization algorithm, which allows to minimizes the sum, over all clusters (small regions), of the within-cluster sums of point-to-cluster-centroid Euclidean distances. In fact, we selected this relative simple clusterization algorithm motivated on the fact that the mouse cerebral cortex presents a clear smoothed convexity (without the presence of pronounced sulcus and gyrus structures like for the brain of other species), which allows to reach a smooth parcellation over each hemispheric cerebral cortex's surface. Finally, the parcellation procedure resulted on a modified WHS parcellation scheme of 75 cortical and subcortical gray matter regions for each hemisphere (for a list of region labels see Table S1). Then, the WHS T2-Weighted MR image was segmentated using SPMMOUSE and SPM5 toolboxes, and resulting non-linear warping transformation parameters obtained were applied to the modified WHS parcellation scheme in order to transforms it into the standard template space of the SPMMOUSE toolbox, similarly as done with the individual background control and shiverer diffusion tensor maps as mentioned above.

### Axonal connectivity estimation

For each subject, axonal trajectories between each pair of gray matter regions (defined by the normalized modified WHS parcellation scheme) were estimated using 3 fully automated fiber tractography algorithms: 1) traditional streamline [26], 2) tensorline [58] and 3) tensor deflection [59]. In the text we refer to these algorithms as: “FACT”, “TL” and “TEND”, respectively. Tracking parameters used were: 25 µm as step size, 200 mm as maximum trace length, ±80° as curvature threshold over voxel, and 0.12 as FA threshold. Seed points were selected as all white matter brain voxels with an FA value greater than 0.12 (the so-called brute-force approach). The previous selection of a relative low FA threshold was carried out with the purpose of do not impose in the experiment an initial difference between the two groups with the selection of a higher FA threshold value (e.g. 0.2), which had provoked an early groups difference on the number of seed points, and subsequently on connectivity density and brain network properties. In this sense, we verified a non-significant groups difference (P = 0.9712) between the number of seed points that satisfied the here imposed condition (FA>0.12), while on the contrary a significant difference (P = 0.0068) was found for the more typically used FA threshold value of 0.2.

### Network construction

For each subject, whole brain undirected weighted networks were created for each of the tracking algorithms as follows: 1) a node was defined to represent each considered anatomic region, 2) an undirected arc a_ij_ between any nodes i and j was established with a corresponding arc weight w(a_ij_), defined as the effective number of connecting fiber trajectories relative to the number of voxels over the surface of regions i and j, where each fiber path was quantified according to the arithmetic mean of the inverse of its mean diffusivity values. Mathematically:



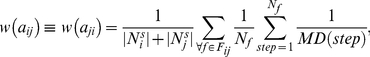
(1)where 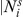 and 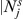 are the number of elements (superficial nodes) of regions *i* and *j* respectively, 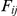 is the set of fiber trajectories connecting regions *i* an *j*, 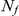 is the number of steps of fiber trajectory 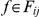, and *MD(step)* the local mean diffusivity of fiber trajectory 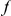 in each *step*. Note that region-region connection arc weights are defined not only taking into account the basic white matter structure but also an indirect measure of the potential efficacy of each nervous fiber pathway (for similar arc weight definitions see [60], where the mean of the inverse of the ADC measure was used for a brain maturation analysis, as well as [5], where the FA measure was used to define arc weight in an Alzheimer's Disease study). In the *Discussion* section (*Methodological Issues and Future Work* subsection) we comment more about this point.

Finally, for each created brain structural network its connectivity backbone was estimated [19]: first, a maximum spanning tree, which connects all nodes of the network such that the sum of its weights is maximal, was extracted; then, additional edges were added in order of their weight until the average node degree was 4. All posterior network analysis and visual representations were based on the resultant networks (connectivity backbones).

### Graph analysis

Each structural whole brain network obtained was characterized attending to six basic metrics:

#### Clustering index (*C*)

A measure of the inherent tendency to cluster nodes into strictly connected neighborhoods. In a weighted graph G, the clustering around a node *i* can be calculated as the geometric average of subgraph node weights [61]:



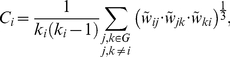
(2)where *k_i_* is the number of arcs connecting node *i* and the weights a*r*e scaled by the largest weight in the network, 
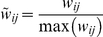
. The clustering coefficient for the whole graph G is defined as the average of clustering around each one of the *n* nodes:



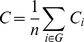
(3)


#### Characteristic path length (*L*)

A measure of the typical separation between any two nodes *i* and *j*, and it is defined as the mean of geodesic lengths 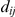 over all pairs of nodes:



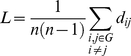
(4)


In the unweighted network context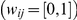, the geodesic length 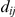 is defined as the number of arcs along the shortest path connecting nodes *i* and *j*. In the case of weighted networks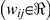, the path with the minimum number of nodes is not necessarily the optimal 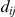 and in some cases it is necessary to define a physical length associated to each arc (this should be a function of the characteristics of the hypothetical link among any nodes *i* and *j*). In this work, we assumed that the physical length of an arc connecting nodes *i* and *j* is inversely proportional to the strength of the analyzed connection [18], i.e. 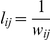. Thus, the shortest path length 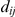 is finally computed as the smallest sum of the arc lengths throughout all the possible paths from node *i* to node *j*. Note that for the particular case of unweighted graphs, 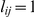 for all arcs and the geodesic lengths 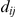 reduces to the minimum number of arcs traversed to get from *i* to *j*.

#### Modularity (*Q*)

A measure of the degree to which a network may be subdivided into modules or communities, reflecting the inherent tendency to the appearance of densely connected groups of vertices with sparser connections between groups [62], [63]. The modularity for a given partition of a network *G* is defined as [63]:



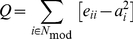
(5)where *N_mod_* is the number of modules, *e_ii_* is the fraction of edges in the network that connect vertices within the community *i*, and *a_i_* represents the fraction of edges that connect vertices of community *i* with other communities (i.e., 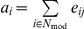). This quantity measures the fraction of the edges in the network that connect vertices of the same type (i.e., within community edges) minus the expected value of the same quantity in a network with the same community divisions but random connections between the vertices. If the number of within-community edges is no better than random, we will get *Q = *0. Values approaching *Q = *1, which is the maximum, indicate networks with strong community structure [63].

In order to identify the modulus of the created structural brain networks that optimize the previous modularity measure (equation 5), we used Newman's spectral optimization method [62] that is implemented as part of the Brain Connectivity Toolbox [64] (available at http://www.brain-connectivity-toolbox.net).

#### Efficiency parameters (*E_glob_, E_loc_*)

In terms of the information flow, the *global efficiency* (E_glob_) of a network G reflects how efficiently information can be exchanged over G, considering a parallel system in which each node sends information concurrently along the network. It is defined as [65]:



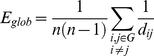
(6)


The *local efficiency* (E_loc_) of G is defined as the average efficiency of the local subgraphs [65]:



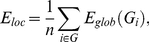
(7)where G_i_ is the subgraph of the first neighbors of node *i*. This measure has been used to reveal how much a system is fault tolerant, indicating how efficient the communication is among the first neighbors of *i* when *i* is removed.

In a physiological sense, the global efficiency of a structural brain network reflects the potential parallel exchange of neural information between the involved anatomic regions (a high global efficiency value, i.e. E_glob_≈1, may indicate highly parallel information transfer in the brain system, in which each element node could efficiently send information concurrently along the network). The local efficiency of a structural brain network reflects its potential tendency to have communities or clusters of anatomically and physiologically different regions that deal with common neural information (where regions connected to a same region tend also to link to each other). In addition, concurrent higher values of global and local efficiency indicate a system with a high balance between local necessities (fault tolerance) and wide-scope interactions.

#### Small-world parameter (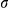)

Small-world networks are defined as those having small mean shortest path length, like random networks (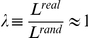), and high clustering coefficient, much larger than random networks (
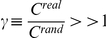
) [66]. Additionally, the small-worldness condition lies in satisfying that 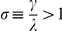 [67]. A network is said to shifts toward a random network if is small-worldness parameter decreases due to gamma (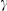) decreases and/or lambda (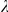) increases.

### Network measures spatial representations

For each network measure we define here its characteristic *representation space*, in which each subject is spatially represented and determined by a unique point attending to its topological properties. Formally, for a given network measure X, we define the N available values of X obtained for each subject *i* as the coordinates of the point that represents subject *i* in the N-dimensional Euclidean space of measure X. Specifically, because here we used three different fiber tracking algorithms, the three values of any measure X obtained for each subject *i* are assumed as the “length”, “width” and “depth” respectively of a point determining uniquely the position of subject *i* on the 3-dimensional space of X. In summary, for each considered brain network measure we obtained a 3-dimensional space in which subjects are represented for unique points whose coordinates corresponds to the obtained values for this measure (see for example Figure 2).

Similarly, the *representation space* of a set of M network measures can be created assuming a point for each subject but, as we have N different values per network measure, now the resulting spatial representation will be on the M*N-dimensional Euclidean space (i.e. an abstract 18-dimensional Euclidean space representing the 6 network measures considered in this study, where to each measure corresponds three coordinates).

### Subjects classification

The potential prediction of structural network measures was assessed using linear discriminant analysis (LDA) [45]. This is an extended classification procedure that assumes a multivariate normal distribution of classes around their means and a common covariance matrix, resulting in a linear classification boundary.

In the context of the *network measure spatial representations*, for each network measure the LDA procedure was employed according to a cross-validation approach, in which all but one mouse (a control or a shiverer) were used for training, and the left-out mouse was used for testing. Then the train/test partition was rotated until all subjects have been tested without being included in the training sample. In summary, for each subject the LDA returned the posterior probability of this subject to belong to each of the two training subgroups (integrated by the rest of the control or shiverer subjects respectively; see for example Table 2), as well as the coefficients of the boundary curves between the groups (i.e. the equations of the hyperplane that best separated the groups in the corresponding 3-dimensional *representation space*).

Finally, for subjects classification based on the combination of the considered 6 network measures, the LDA procedure was similar employed according to a cross-validation approach but in this case the number of features (dimensionality = 6) was previously reduced by means of a forward sequential feature selection in a wrapper fashion [46]. In general, the forward sequential feature selection in a wrapper fashion selects a subset of features by sequentially adding a new feature (forward search) until certain stopping conditions are satisfied. More specifically, the feature selection procedure starts prediction using only the network measure that resulted the best single predictor (usually the clustering index or the small-worldness parameter), after this initial prediction, the procedure adds as a new predictor (feature) to the network measure that resulted the second best single predictor, and continues in a similar way (the order of the network measures inclusion can be easily deduced from Table 2, on *Results* section) until the inclusion of a new network measure do not implies an improvement in prediction. According to this procedure, final discrimination results were based on the interaction of the most relevant features, where redundant information is considerable reduced.

### Statistical analysis

In order to evaluate differences between two groups of subjects for a same network measure we used a permutation test. This has the following advantages: the test is distribution free, no assumptions of an underlying correlation structure are required, and provides exact p-values for any number of subjects and estimation algorithms. Specifically, for each network the statistics t and *max t* were calculated, where *max t* represent the maximum of statistic t in each fiber tracking algorithm result. The distribution estimated by permutation techniques for *max t* was then used to set significance levels that control the experiment wise error for the simultaneous univariate comparisons [68], [69].

## Supporting Information

Table S1 **Mouse considered regions for each brain hemisphere (modified WHS parcellation scheme).**
(DOC)

Table S2
**Gamma (** 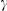 **) and lambda (** 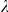 **) parameters obtained for the brain anatomical networks of control and shiverer mice groups. For each measure and fiber tracking algorithm, mean values are reported with their corresponding standard errors (i.e. the uncertainty of how the sample mean represents the underlying population mean).** For each measure, the multivariate permutation P-value corresponds to the null hypothesis that medians of obtained group values are equal (a P-value near to zero, i.e. P < 0.05, indicates a significant difference between groups). The small P-value obtained for 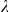 indicates a significant increase on the shiverer subjects of this structural network attribute, whereas the 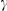 parameter doesn't show significant differences (although a non significant decrease can be noted). This result, together with the reported significant decrease for the 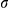 parameter, supports the hypothesis of a structural brain network randomization in the shiverer mutant mouse.(DOC)

Table S3
**Individual conditioned probabilities of being a control subject with regard clustering (***C***** **), characteristic path length (***L***** **), modularity (** ***Q*** **), global efficiency (** ***E_glob_*** **), local efficiency (** ***E_loc_*** **) or small-worldness (** 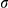 **) measures obtained for the brain anatomical networks (estimated in individual native spaces) of control and shiverer mice subjects (preceded by the prefixes Wt and Shi, respectively).** For each subject, a P(Cs|I_i_) value near to one, e.g. P > 0.95, indicates a high probability of belonging to the control group according to the structural network measure I_i_; whereas a P(Cs|I_i_) value near to zero, e.g. P < 0.05, indicates a high probability of belonging to the shiverer group. For comparison, corresponding conditioned probability of being a shiverer subject according to I_i_ can be obtained similarly as 1-P(Cs|I_i_). For each measure, or the combination of all them, the Correct Prediction value indicates the % of subjects that were correctly classified. Note how predictions accuracy, for each considered network measure or the combination of all them, decreases considerably with regard the corresponding results obtained in the standard template space (Table 2 on *Results* section), which supports the point of view that in the case of DW-MRI techniques, subjects transformation to a standard space allows the improvement of statistical brain network comparisons by reducing variability on networks estimations resultant from technical limitations.(DOC)

Table S4
**Individual conditioned probabilities of being a control subject with regard clustering (** ***C*** **), characteristic path length (** ***L*** **), modularity (** ***Q*** **), global efficiency (** ***E_glob_*** **), local efficiency (** ***E_loc_*** **) or small-worldness (** 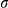 **) measures obtained for the brain anatomical networks (using mean FA as an indicator of each fiber path's integrity) of control and shiverer mice subjects (preceded by the prefixes Wt and Shi, respectively).** For each subject, a P(Cs|I_i_) value near to one, e.g. P > 0.95, indicates a high probability of belonging to the control group according to the structural network measure I_i_; whereas a P(Cs|I_i_) value near to zero, e.g. P < 0.05, indicates a high probability of belonging to the shiverer group. For comparison, corresponding conditioned probability of being a shiverer subject according to I_i_ can be obtained similarly as 1-P(Cs|I_i_). For each measure, or the combination of all them, the Correct Prediction value indicates the % of subjects that were correctly classified. Note that although in general prediction accuracies are considerable high, particularly for *C*, *E_glob_*, *E_loc_* and the combination of the 6 considered network measures (i.e. 91.66 % of prediction accuracy), the obtained values are lower than those obtained when the mean value of the inverse of MD was used to define arcs weights (Table 2 on *Results* section).(DOC)

Table S5
**Individual conditioned probabilities of being a control subject with regard clustering (** ***C*** **), characteristic path length (** ***L*** **), modularity (** ***Q*** **), global efficiency (***E_glob_***** **), local efficiency (***E_loc_***** **) or small-worldness (** 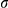 **) measures obtained for the brain anatomical networks (with arc weights between nodes [regions] defined only as the number of connecting fiber paths, i.e., without any indicator of fiber integrity) of control and shiverer mice subjects (preceded by the prefixes Wt and Shi, respectively).** For each subject, a P(Cs|I_i_) value near to one, e.g. P > 0.95, indicates a high probability of belonging to the control group according to the structural network measure I_i_; whereas a P(Cs|I_i_) value near to zero, e.g. P < 0.05, indicates a high probability of belonging to the shiverer group. For comparison, corresponding conditioned probability of being a shiverer subject according to I_i_ can be obtained similarly as 1-P(Cs|I_i_). For each measure, or the combination of all them, the Correct Prediction value indicates the % of subjects that were correctly classified. Note how predictions accuracy, for each considered network measure or the combination of all them, decreases considerably with regard the corresponding results obtained when the mean value of the inverse of MD or the mean FA value were used as measures of fiber integrity (see Table 2 and Table S5, respectively).(DOC)

Table S6
**Pearson correlations values between the six topological measures obtained for brain anatomical networks of mice subjects: clustering (***C***** **), characteristic path length (***L***** **), modularity (***Q***** **), global efficiency (***E_glob_***** **), local efficiency (***E_loc_***** **) and small-worldness (** 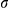 **).** For the sake of simplicity, here we present correlations only trough measures (i.e. without taking into account differences between groups or fiber tracking algorithms). Asterisks indicate significant correlations (i.e. whit a corresponding P<0.05). Note that almost all pairs of measures are significantly correlated (except pairs *C-L, L-Q, L- E_loc_, Q- E_glob_* and *Q-* 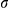), illustrating the need of reduce redundant network features information when two o more measures are combined with the purpose of obtain a final quantitative subject discrimination.(DOC)

Table S7 **Prediction accuracies (%) obtained for control and shiverer mice subjects according to results of each fiber tracking algorithm and the combination of all them, with regard clustering (** ***C*** **), characteristic path length (** ***L*** **), modularity (** ***Q*** **), global efficiency (** ***E_glob_*** **), local efficiency (** ***E_loc_*** **) or/and small-worldness (** ***σ*** **) brain anatomical network measures.** For each network measure and fiber tracking, or the combination of all them, the Prediction accuracy indicates the % of subjects that were correctly classified. Note that the combination of various fiber tracking algorithms contributes to the stabilization and consistency of the classification results, in other words, a high prediction for a given network measure usually coincides with a high prediction for the other measures, which do not happens always for the results corresponding to only a given fiber tracking algorithm. For example, in the case of the TL algorithm, although a high prediction accuracy (around 91.66∼100%) was obtained for *C*, *E_glob_*, *σ* and the combination of the six network measures, the prediction accuracy obtained for *E_loc_* (i.e. 83.33%) was considerable lower than the obtained for the others algorithms and the combination of the three algorithms (around 91.66∼100%); similarly happened with predictions obtained from FACT and TEND algorithms for *σ* and the combination of the six network measures. These results support the point of view that the use of different tractography algorithms makes the results robust to choice of tracking algorithm, which is potentially a significant source of bias.
(DOC)
