# Supplementary material for: Automated Discrimination of Brain Pathological State Attending to Complex Structural Brain Network Properties: The Shiverer Mutant Mouse Case
Source: PLoS One. 2011 May 27;6(5):e19071. doi: 10.1371/journal.pone.0019071 (PMC3103505; doi:10.1371/journal.pone.0019071)
Supplement: Table S1 — Mouse considered regions for each brain hemisphere (modified WHS parcellation scheme). (DOC) [file pone.0019071.s001.doc]

| **No.** | **Label** |
| --- | --- |
| 1 | Thalamus |
| 2 | Superior Colliculus |
| 3 | Inferior Colliculus |
| 4 | Lateral lemniscus |
| 5 | Periaqueductal gray |
| 6 | Septal nuclei complex lateral |
| 7 | Ventral nuclei of the thalamus |
| 8 | Pontine gray |
| 9 | Substantia nigra |
| 10 | Interpeduncular nucleus |
| 11 | Globus pallidus |
| 12 | Deep mesencephalic nuclei |
| 13 | Lateral dorsal nucleus of thalamus |
| 14 | Medial Geniculate |
| 15 | Anterior pretectal nucleus |
| 16 | Striatum |
| 17 | Hippocampus |
| 18 | Lateral Geniculate |
| 19 | Pineal Gland |
| 20 | Amygdala |
| 21 | Hypothalamus |
| 22 | Nucleus accumbens |
| 23 | Olfactory areas |
| 24 | Cochlear nuclei |
| 25 | Cerebellum |
| 26-75 | Small cortical regions of  approximately the same volume (1.66±0.23 mm^3^) |
