# Supplementary material for: Automated Discrimination of Brain Pathological State Attending to Complex Structural Brain Network Properties: The Shiverer Mutant Mouse Case
Source: PLoS One. 2011 May 27;6(5):e19071. doi: 10.1371/journal.pone.0019071 (PMC3103505; doi:10.1371/journal.pone.0019071)
Supplement: Table S2 — Gamma ( ) and lambda ( ) parameters obtained for the brain anatomical networks of control and shiverer mice groups. For each measure and fiber tracking algorithm, mean values are reported with their corresponding standard errors (i.e. the uncertainty of how the sample mean represents the underlying population mean). For each measure, the multivariate permutation P-value corresponds to the null hypothesis that medians of obtained group values are equal (a P-value near to zero, i.e. P < 0.05, indicates a significant difference between groups). The small P-value obtained for indicates a significant increase on the shiverer subjects of this structural network attribute, whereas the parameter doesn't show significant differences (although a non significant decrease can be noted). This result, together with the reported significant decrease for the parameter, supports the hypothesis of a structural brain network randomization in the shiverer mutant mouse. (DOC) [file pone.0019071.s002.doc]

| **Group** | | **Brain network measures**  **(Mean ± SEM)** | |
| --- | --- | --- | --- |
|  |  | $\gamma$ | $\lambda$ |
| Control | FACT | 7.6285±0.1863 | 1.3985±0.0382 |
|  | TL | 8.1151±0.2790 | 1.3112±0.0347 |
|  | TEND | 9.2655±0.1940 | 1.5791±0.0462 |
| Shiverer | FACT | 7.5311±0.1813 | 1.4210±0.0923 |
|  | TL | 7.5458±0.3092 | 1.9842±0.2001 |
|  | TEND | 8.9363±0.2368 | 1.8108±0.1318 |
| **P-value** | | 0.3621 | **0.0084** |
