# Supplementary material for: Automated Discrimination of Brain Pathological State Attending to Complex Structural Brain Network Properties: The Shiverer Mutant Mouse Case
Source: PLoS One. 2011 May 27;6(5):e19071. doi: 10.1371/journal.pone.0019071 (PMC3103505; doi:10.1371/journal.pone.0019071)
Supplement: Table S3 — Individual conditioned probabilities of being a control subject with regard clustering (C ), characteristic path length (L ), modularity ( Q ), global efficiency ( Eglob ), local efficiency ( Eloc ) or small-worldness ( ) measures obtained for the brain anatomical networks (estimated in individual native spaces) of control and shiverer mice subjects (preceded by the prefixes Wt and Shi, respectively). For each subject, a P(Cs|Ii) value near to one, e.g. P > 0.95, indicates a high probability of belonging to the control group according to the structural network measure Ii; whereas a P(Cs|Ii) value near to zero, e.g. P < 0.05, indicates a high probability of belonging to the shiverer group. For comparison, corresponding conditioned probability of being a shiverer subject according to Ii can be obtained similarly as 1-P(Cs|Ii). For each measure, or the combination of all them, the Correct Prediction value indicates the % of subjects that were correctly classified. Note how predictions accuracy, for each considered network measure or the combination of all them, decreases considerably with regard the corresponding results obtained in the standard template space (Table 2 on Results section), which supports the point of view that in the case of DW-MRI techniques, subjects transformation to a standard space allows the improvement of statistical brain network comparisons by reducing variability on networks estimations resultant from technical limitations. (DOC) [file pone.0019071.s003.doc]

| **Subjects** | P(Cs|*C*) | P(Cs|*L*) | P(Cs|*Q*) | P(Cs|*E_glob_*) | P(Cs|*E_loc_*) | P(Cs|$\sigma$) | P(Cs|*C,L,A,*  *E_glob_,E_loc_,*$\sigma$) |
| --- | --- | --- | --- | --- | --- | --- | --- |
| Wt 1 | 0.9999 | 0.4526 | 0.2851 | 0.9998 | 0.9961 | 0.3433 | 0.9999 |
| Wt 2 | 0.9635 | 0.6170 | 0.0370 | 0.9805 | 0.0285 | 0.0808 | 0.4369 |
| Wt 3 | 0.9991 | 0.3430 | 0.5124 | 0.7836 | 0.9999 | 0.4146 | 0.9999 |
| Wt 4 | 0.9819 | 0.6063 | 0.0611 | 0.0001 | 0.9951 | 0.6604 | 0.9999 |
| Wt 5 | 0.9995 | 0.6189 | 0.7637 | 0.9988 | 0.9000 | 0.6040 | 0.9999 |
| Wt 6 | 0.9994 | 0.7850 | 0.6607 | 0.9996 | 0.9578 | 0.5502 | 0.9999 |
| Shi 1 | 0.9896 | 0.9985 | 0.3554 | 0.9999 | 0.4954 | 0.8943 | 0.9894 |
| Shi 2 | 0.0010 | 0.2471 | 0.4636 | 0.0013 | 0.4969 | 0.3560 | 0.0010 |
| Shi 3 | 1.53e-05 | 0.1950 | 0.5402 | 0.0069 | 0.0050 | 0.7937 | 7.75e-08 |
| Shi 4 | 0.0054 | 0.4962 | 0.7487 | 0.0187 | 0.0741 | 0.3480 | 0.0004 |
| Shi 5 | 1.90e-09 | 0.9995 | 0.3931 | 0.0002 | 0.0007 | 0.6360 | 1.46e-12 |
| Shi 6 | 0.0002 | 0.2670 | 0.3389 | 0.0004 | 0.0033 | 0.3980 | 6.77e-07 |
| **Predicted (%)** | 91.67 | 66.67 | 58.33 | 83.33 | 91.67 | 50 | 83.33 |
