# Supplementary material for: Automated Discrimination of Brain Pathological State Attending to Complex Structural Brain Network Properties: The Shiverer Mutant Mouse Case
Source: PLoS One. 2011 May 27;6(5):e19071. doi: 10.1371/journal.pone.0019071 (PMC3103505; doi:10.1371/journal.pone.0019071)
Supplement: Table S4 — Individual conditioned probabilities of being a control subject with regard clustering ( C ), characteristic path length ( L ), modularity ( Q ), global efficiency ( Eglob ), local efficiency ( Eloc ) or small-worldness ( ) measures obtained for the brain anatomical networks (using mean FA as an indicator of each fiber path's integrity) of control and shiverer mice subjects (preceded by the prefixes Wt and Shi, respectively). For each subject, a P(Cs|Ii) value near to one, e.g. P > 0.95, indicates a high probability of belonging to the control group according to the structural network measure Ii; whereas a P(Cs|Ii) value near to zero, e.g. P < 0.05, indicates a high probability of belonging to the shiverer group. For comparison, corresponding conditioned probability of being a shiverer subject according to Ii can be obtained similarly as 1-P(Cs|Ii). For each measure, or the combination of all them, the Correct Prediction value indicates the % of subjects that were correctly classified. Note that although in general prediction accuracies are considerable high, particularly for C, Eglob, Eloc and the combination of the 6 considered network measures (i.e. 91.66 % of prediction accuracy), the obtained values are lower than those obtained when the mean value of the inverse of MD was used to define arcs weights (Table 2 on Results section). (DOC) [file pone.0019071.s004.doc]

| **Subjects** | P(Cs|*C*) | P(Cs|*L*) | P(Cs|*Q*) | P(Cs|*E_glob_*) | P(Cs|*E_loc_*) | P(Cs|$\sigma$) | P(Cs|*C,L,A,*  *E_glob_,E_loc_,*$\sigma$) |
| --- | --- | --- | --- | --- | --- | --- | --- |
| Wt 1 | 0.9999 | 0.0018 | 0.5500 | 0.9999 | 0.9999 | 0.9999 | 0.9999 |
| Wt 2 | 0.3276 | 0.4898 | 0.7333 | 0.4022 | 0.6888 | 0.7456 | 0.5190 |
| Wt 3 | 0.9997 | 0.5946 | 0.7166 | 0.9753 | 0.9981 | 0.3953 | 0.9999 |
| Wt 4 | 0.9973 | 0.6162 | 0.5500 | 0.9966 | 0.9976 | 0.3820 | 0.9999 |
| Wt 5 | 0.9882 | 0.5048 | 0.6333 | 0.9991 | 0.9874 | 0.8162 | 0.9998 |
| Wt 6 | 0.9790 | 0.2971 | 0.6333 | 0.9992 | 0.8694 | 0.8422 | 0.9967 |
| Shi 1 | 0.0265 | 0.8140 | 0.4500 | 0.0189 | 0.0382 | 0.5327 | 0.0010 |
| Shi 2 | 0.0018 | 0.6770 | 0.3666 | 8.27e-05 | 0.0003 | 0.0363 | 7.51-07 |
| Shi 3 | 0.4383 | 0.3915 | 0.4500 | 0.2727 | 0.9593 | 0.0669 | 0.9485 |
| Shi 4 | 0.0006 | 0.5508 | 0.7333 | 0.0051 | 0.0062 | 0.5059 | 4.18e-06 |
| Shi 5 | 5.28e-06 | 0.5510 | 0.8166 | 0.0001 | 8.20e-08 | 0.1657 | 4.34e-13 |
| Shi 6 | 0.0017 | 0.4485 | 0.7166 | 0.001 | 0.0064 | 0.2478 | 1.15e-05 |
| **Predicted (%)** | 91.66 | 41.66 | 41.66 | 91.66 | 91.66 | 66.66 | 91.66 |
