# Supplementary material for: Automated Discrimination of Brain Pathological State Attending to Complex Structural Brain Network Properties: The Shiverer Mutant Mouse Case
Source: PLoS One. 2011 May 27;6(5):e19071. doi: 10.1371/journal.pone.0019071 (PMC3103505; doi:10.1371/journal.pone.0019071)
Supplement: Table S5 — Individual conditioned probabilities of being a control subject with regard clustering ( C ), characteristic path length ( L ), modularity ( Q ), global efficiency (Eglob ), local efficiency (Eloc ) or small-worldness ( ) measures obtained for the brain anatomical networks (with arc weights between nodes [regions] defined only as the number of connecting fiber paths, i.e., without any indicator of fiber integrity) of control and shiverer mice subjects (preceded by the prefixes Wt and Shi, respectively). For each subject, a P(Cs|Ii) value near to one, e.g. P > 0.95, indicates a high probability of belonging to the control group according to the structural network measure Ii; whereas a P(Cs|Ii) value near to zero, e.g. P < 0.05, indicates a high probability of belonging to the shiverer group. For comparison, corresponding conditioned probability of being a shiverer subject according to Ii can be obtained similarly as 1-P(Cs|Ii). For each measure, or the combination of all them, the Correct Prediction value indicates the % of subjects that were correctly classified. Note how predictions accuracy, for each considered network measure or the combination of all them, decreases considerably with regard the corresponding results obtained when the mean value of the inverse of MD or the mean FA value were used as measures of fiber integrity (see Table 2 and Table S5, respectively). (DOC) [file pone.0019071.s005.doc]

| **Subjects** | P(Cs|*C*) | P(Cs|*L*) | P(Cs|Q) | P(Cs|*E_glob_*) | P(Cs|*E_loc_*) | P(Cs|$\sigma$) | P(Cs|*C,L,A,*  *E_glob_,E_loc_,*$\sigma$) |
| --- | --- | --- | --- | --- | --- | --- | --- |
| Wt 1 | 0.9980 | 0.0061 | 0.7333 | 0.9846 | 0.8853 | 0.5924 | 0.9997 |
| Wt 2 | 0.0990 | 0.1493 | 0.7333 | 0.4629 | 0.4961 | 0.0829 | 0.0977 |
| Wt 3 | 0.9982 | 0.6180 | 0.6333 | 0.7243 | 0.9759 | 0.0233 | 0.9999 |
| Wt 4 | 0.9999 | 0.6801 | 0.7333 | 0.9492 | 0.9830 | 0.6131 | 0.9999 |
| Wt 5 | 0.2956 | 0.6240 | 0.6333 | 0.7690 | 0.2538 | 0.3051 | 0.1249 |
| Wt 6 | 0.6402 | 0.4973 | 0.8333 | 0.9955 | 0.9568 | 0.6537 | 0.9752 |
| Shi 1 | 0.1453 | 0.8307 | 0.6333 | 0.2550 | 0.4085 | 0.5469 | 0.1051 |
| Shi 2 | 0.0015 | 0.4867 | 0.3666 | 0.0015 | 0.0095 | 0.2649 | 1.45e-05 |
| Shi 3 | 0.5698 | 0.4241 | 0.4500 | 0.9720 | 0.8674 | 0.5087 | 0.8966 |
| Shi 4 | 0.0116 | 0.8272 | 0.2833 | 0.7496 | 0.3250 | 0.9508 | 0.0056 |
| Shi 5 | 0.0020 | 0.3666 | 0.8166 | 0.0003 | 0.0001 | 0.4297 | 2.90e-07 |
| Shi 6 | 0.1719 | 0.4514 | 0.6333 | 0.3222 | 0.2188 | 0.5503 | 0.0549 |
| **Predicted (%)** | 75 | 58.33 | 50 | 75 | 75 | 41.66 | 75 |
