# Supplementary material for: Automated Discrimination of Brain Pathological State Attending to Complex Structural Brain Network Properties: The Shiverer Mutant Mouse Case
Source: PLoS One. 2011 May 27;6(5):e19071. doi: 10.1371/journal.pone.0019071 (PMC3103505; doi:10.1371/journal.pone.0019071)
Supplement: Table S6 — Pearson correlations values between the six topological measures obtained for brain anatomical networks of mice subjects: clustering (C ), characteristic path length (L ), modularity (Q ), global efficiency (Eglob ), local efficiency (Eloc ) and small-worldness ( ). For the sake of simplicity, here we present correlations only trough measures (i.e. without taking into account differences between groups or fiber tracking algorithms). Asterisks indicate significant correlations (i.e. whit a corresponding P<0.05). Note that almost all pairs of measures are significantly correlated (except pairs C-L, L-Q, L- Eloc, Q- Eglob and Q- ), illustrating the need of reduce redundant network features information when two o more measures are combined with the purpose of obtain a final quantitative subject discrimination. (DOC) [file pone.0019071.s006.doc]

| **Measures** | *C* | *L* | *Q* | *E_glob_* | *E_loc_* | $\sigma$ |
| --- | --- | --- | --- | --- | --- | --- |
| *C* | - | -0.2932 | 0.6582* | 0.6238* | 0.9822* | 0.4934* |
| *L* |  | - | 0.0744 | -0.4674* | -0.2761 | -0.3754* |
| *Q* |  |  | - | 0.0630 | 0.6808* | 0.0786 |
| *E_glob_* |  |  |  | - | 0.5400* | 0.5420* |
| *E_loc_* |  |  |  |  | - | 0.4243* |
| $\sigma$ |  |  |  |  |  | - |
