# Supplementary material for: Automated Discrimination of Brain Pathological State Attending to Complex Structural Brain Network Properties: The Shiverer Mutant Mouse Case
Source: PLoS One. 2011 May 27;6(5):e19071. doi: 10.1371/journal.pone.0019071 (PMC3103505; doi:10.1371/journal.pone.0019071)
Supplement: Table S7 — Prediction accuracies (%) obtained for control and shiverer mice subjects according to results of each fiber tracking algorithm and the combination of all them, with regard clustering ( C ), characteristic path length ( L ), modularity ( Q ), global efficiency ( Eglob ), local efficiency ( Eloc ) or/and small-worldness ( σ ) brain anatomical network measures. For each network measure and fiber tracking, or the combination of all them, the Prediction accuracy indicates the % of subjects that were correctly classified. Note that the combination of various fiber tracking algorithms contributes to the stabilization and consistency of the classification results, in other words, a high prediction for a given network measure usually coincides with a high prediction for the other measures, which do not happens always for the results corresponding to only a given fiber tracking algorithm. For example, in the case of the TL algorithm, although a high prediction accuracy (around 91.66∼100%) was obtained for C, Eglob, σ and the combination of the six network measures, the prediction accuracy obtained for Eloc (i.e. 83.33%) was considerable lower than the obtained for the others algorithms and the combination of the three algorithms (around 91.66∼100%); similarly happened with predictions obtained from FACT and TEND algorithms for σ and the combination of the six network measures. These results support the point of view that the use of different tractography algorithms makes the results robust to choice of tracking algorithm, which is potentially a significant source of bias. (DOC) [file pone.0019071.s007.doc]

| **Prediction (%)** | | *C* | *L* | Q | *E_glob_* | *E_loc_* | *σ* | *C,L,A,*  *E_glob_,E_loc_,σ* |
| --- | --- | --- | --- | --- | --- | --- | --- | --- |
|  | FACT | 91.66 | 58.33 | 83.33 | 91.66 | 91.66 | 33.33 | 91.66 |
|  | TL | 100 | 66.67 | 58.33 | 91.66 | 83.33 | 100 | 100 |
|  | TEND | 91.66 | 83.33 | 8.333 | 91.66 | 91.66 | 75 | 91.66 |
|  | FACT,TL, TEND | 100 | 66.67 | 66.67 | 91.66 | 100 | 100 | 100 |
